# Resolution of Dialyzer Membrane-Associated Thrombocytopenia with Use of Cellulose Triacetate Membrane: A Case Report

**DOI:** 10.1155/2011/134295

**Published:** 2011-04-10

**Authors:** Feyisayo Olafiranye, Win Kyaw, Oladipupo Olafiranye

**Affiliations:** ^1^Department of Family Medicine, State University of New York Downstate Medical Center, 450 Clarkson Avenue, P.O. Box 1199, Brooklyn, NY 11203-2098, USA; ^2^Division of Nephrology, State University of New York Downstate Medical Center, Brooklyn, NY 11203-2098, USA; ^3^Department of Medicine, State University of New York Downstate Medical Center, Brooklyn, NY 11203-2098, USA

## Abstract

Blood and dialyzer membrane interaction can cause significant thrombocytopenia through the activation of complement system. The extent of this interaction determines the biocompatibility of the membrane. Although the newer synthetic membranes have been shown to have better biocompatibility profile than the cellulose-based membranes, little is known about the difference in biocompatibility between synthetic membrane and modified cellulose membrane. Herein, we report a case of a patient on hemodialysis who developed dialyzer-membrane-related thrombocytopenia with use of synthetic membrane (F200NR polysulfone). The diagnosis of dialyzer membrane-associated thrombocytopenia was suspected by the trend of platelet count before and after dialysis, and the absence of other possible causes of thrombocytopenia. We observed significant improvement in platelet count when the membrane was changed to modified cellulose membrane (cellulose triacetate). In patients at high risk for thrombocytopenia, the modified cellulose membrane could be a better alternative to the standard synthetic membranes during hemodialysis.

## 1. Introduction

Thrombocytopenia is common in patients receiving hemodialysis, and it is usually secondary to use of heparin, uremia, sepsis, blood loss, and bone marrow suppression [1]. However, studies have shown that the dialysis membrane itself can cause significant thrombocytopenia through the activation of the complement system [2]. The degree to which the dialyzer membrane activates the complement system determines its biocompatibility. Hemodialysis initially relied predominantly on the use of cellulose-based membranes with poor biocompatibility profile and associated thrombocytopenia and leucopenia [3]. Modification of the cellulose membranes has greatly reduced these complications [4]. 

In recent time, membranes manufactured from synthetic polymers are commonly used because of their superior biocompatibility as compared to cellulose-based membranes. However, whether the biocompatibility of synthetic membrane is superior to that of synthetically modified cellulose membrane remains unclear. Herein, we report a case of a 54-year-old female that developed dialyzer membrane-associated thrombocytopenia while on hemodialysis with synthetic membrane. The platelet count significantly increased when the membrane was changed to modified cellulose.

## 2. Case Report

A 54-year-old female with history of longstanding hypertension, diabetes, and hyperlipidemia, was admitted to the hospital for persistent nausea and vomiting. She reported taking nifedipine, lisinopril, clonidine, insulin, lipitor, and aspirin at home. Initial blood testing showed white blood count of 4.4 × 10^3^ (normal: 3.8–10.8 × 10^3^/UL), hemoglobin of 10.2 g/dL (normal: 13.2–17.1 g/dL), platelet of 88 × 10^3^/UL (normal: 140–400 × 10^3^/UL), blood urea nitrogen of 42 mg/dL (normal: 7–25 mg/dL), and creatinine of 5.8 mg/dL (normal: 0.7–1.3 mg/dL). Other laboratory tests were within normal limits.

**Figure 1:**
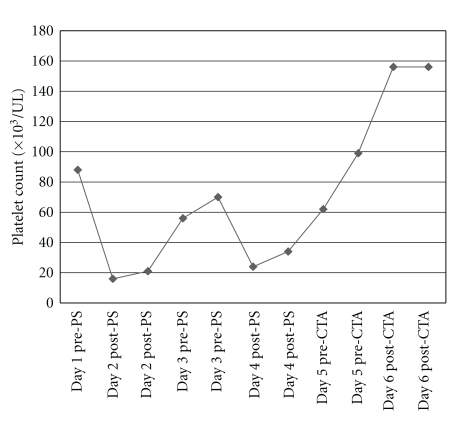
The trend of platelet counts before and after hemodialysis with polysulfone (PS) and cellulose triacetate (CTA) dialyzer membrane.

Dialysis was initiated for end-stage renal disease according to the following prescription: dialyzer, Fresenius Optiflux F200NR (polysulfones synthetic dialyzer membrane, 2.0 m^2^ surface area, and electron-beam sterilized); treatment time: 3 hours; no heparin was given because of baseline mild thrombocytopenia. The treatment was well tolerated. Postdialysis platelet count dropped to 16,000/UL after the first dialysis session. The patient reported no episodes of bleeding or bruising. Repeat platelet count was the same. Peripheral smear showed few platelets and giant cells with no schistocytes. Blood testing for heparin-induced thrombocytopenia antibodies and cultures was negative. Serial platelet counts were done for close monitoring and to determine the need for transfusion. Gradual drop, then rise in platelet count to baseline was noted over a 48-hr period. Following the second dialysis session, similar pattern of fall and rise in platelet count was noticed as shown in Figure 1. However both the hemoglobin and white blood cell count remained stable. The predialysis rise and postdialysis fall in platelet count raised the suspicion for dialysis-related thrombocytopenia. At the third dialysis session, dialyzer membrane was changed to Baxter cellulose triacetate exceltra 210 (modified cellulose dialyzer membrane, 2.1 m^2^ surface area, gamma sterilized) and the platelet count remained stable after dialysis. 

## 3. Discussion

The interaction between blood constituents and dialyzer membrane is increasingly being recognized as a major cause of thrombocytopenia in dialysis patients [2]. The extent of these interactions is related to the composition and geometry of the membrane. Although the cellulose-based membranes are less expensive, synthetic membranes are often used because of their superior biocompatibility [5]. 

Cellulose membranes are composed of polysaccharide units with hydroxyl groups which are responsible for the biochemical interactions and associated complications through the activation of the complement system. However, they are less likely to provoke platelet activation and adhesion [6]. Modifications of these membranes by replacing the hydroxyl groups with acetyl groups have been shown to improve the biocompatibility profile [7]. Synthetic membranes are made up of hydrophobic materials with low tendency to activate the complement system [6]. However, they are more likely to trigger platelet adhesion and thrombus formation which may account for the observed decrease in platelet count each time F200NR polysulfone synthetic dialyzer was used in our patient. The improvement in platelet count following the change of dialyzer membrane to cellulose triacetate membrane may also be related to the reported improved biocompatibility profile of modified cellulose membranes [7]. The baseline thrombocytopenia prior to the initiation of hemodialysis in our patient was most likely due to uremia itself, given the negative workup for common causes of thrombocytopenia. 

Among 96 patients with thrombocytopenia and acute kidney injury requiring continuous venovenous hemofiltration, significant drop in platelet count was observed in the polysulfone without anticoagulation group but not in the cellulose triacetate without anticoagulation group [8]. This finding is consistent with the observation in our patient. In another study comparing the influence of cellulose triacetate (CTA) and polysulfone (PS) membrane on platelet activation, the level of bound GPIIb/IIIa was significantly higher in the polysulfone group [9]. The present case report is consistent with the findings of these studies and further highlights the usefulness of the cheap cellulose-based membrane in patients at high risk for thrombocytopenia. The main clue to the diagnosis of dialyzer-membrane-induced thrombocytopenia in this patient was the trend of platelet counts and absence of other common causes of thrombocytopenia. We are not aware of any prior similar case report.

## 4. Conclusion

In patients at high risk for thrombocytopenia during hemodialysis, the modified cellulose membranes could be a better alternative to the standard synthetic membranes in preventing dialyzer membrane-associated thrombocytopenia. Peridialysis pattern of platelet counts may provide a clue to the diagnosis of dialysis-associated thrombocytopenia.
